# Differential Effects of an O-GlcNAcase Inhibitor on Tau Phosphorylation

**DOI:** 10.1371/journal.pone.0035277

**Published:** 2012-04-19

**Authors:** Yang Yu, Lan Zhang, Xiaojing Li, Xiaoqin Run, Zhihou Liang, Yi Li, Ying Liu, Moon H. Lee, Inge Grundke-Iqbal, Khalid Iqbal, David J. Vocadlo, Fei Liu, Cheng-Xin Gong

**Affiliations:** 1 Department of Neurochemistry, New York State Institute for Basic Research in Developmental Disabilities, Staten Island, New York, United States of America; 2 Department of Developmental Neurobiology, New York State Institute for Basic Research in Developmental Disabilities, Staten Island, New York, United States of America; 3 Department of Molecular Biology and Biochemistry, Simon Fraser University, Burnaby, British Columbia, Canada; The University of Sydney, Australia

## Abstract

Abnormal hyperphosphorylation of microtubule-associated protein tau plays a crucial role in neurodegeneration in Alzheimer's disease (AD). The aggregation of hyperphosphorylated tau into neurofibrillary tangles is also a hallmark brain lesion of AD. Tau phosphorylation is regulated by tau kinases, tau phosphatases, and O-GlcNAcylation, a posttranslational modification of proteins on the serine or threonine residues with β-N-acetylglucosamine (GlcNAc). O-GlcNAcylation is dynamically regulated by O-GlcNAc transferase, the enzyme catalyzing the transfer of GlcNAc to proteins, and N-acetylglucosaminidase (OGA), the enzyme catalyzing the removal of GlcNAc from proteins. Thiamet-G is a recently synthesized potent OGA inhibitor, and initial studies suggest it can influence O-GlcNAc levels in the brain, allowing OGA inhibition to be a potential route to altering disease progression in AD. In this study, we injected thiamet-G into the lateral ventricle of mice to increase O-GlcNAcylation of proteins and investigated the resulting effects on site-specific tau phosphorylation. We found that acute thiamet-G treatment led to a decrease in tau phosphorylation at Thr181, Thr212, Ser214, Ser262/Ser356, Ser404 and Ser409, and an increase in tau phosphorylation at Ser199, Ser202, Ser396 and Ser422 in the mouse brain. Investigation of the major tau kinases showed that acute delivery of a high dose of thiamet-G into the brain also led to a marked activation of glycogen synthase kinase-3β (GSK-3β), possibly as a consequence of down-regulation of its upstream regulating kinase, AKT. However, the elevation of tau phosphorylation at the sites above was not observed and GSK-3β was not activated in cultured adult hippocampal progenitor cells or in PC12 cells after thiamet-G treatment. These results suggest that acute high-dose thiamet-G injection can not only directly antagonize tau phosphorylation, but also stimulate GSK-3β activity, with the downstream consequence being site-specific, bi-directional regulation of tau phosphorylation in the mammalian brain.

## Introduction

Microtubule-associated protein tau is a cytosolic protein that stimulates microtubule assembly and stabilizes microtubule structure. The integrity of the microtubule system is essential for the transport of materials between the cell body and synaptic terminals of neurons. The microtubule system is disrupted and replaced by the accumulation of highly phosphorylated tau as neurofibrillary tangles in affected neurons in the brains of individuals with Alzheimer disease (AD) and other neurodegenerative disorders collectively called tauopathies. Neurofibrillary tangles are also one of the hallmark histopathological lesions of AD brain. Many studies have demonstrated the critical role of hyperphosphorylation and aggregation of tau in neurodegeneration in AD and other tauopathies. The abnormal hyperphosphorylation may cause dissociation of tau from microtubules and, consequently, raise intracellular tau concentration enough to initiate its polymerization into neurofibrillary tangles [1].

The mechanisms by which tau becomes abnormally hyperphosphorylated in AD and other tauopathies are not well understood. Many studies have demonstrated that in the brain, tau phosphorylation is mainly controlled by the kinases glycogen synthase kinase-3β (GSK-3β) and cyclin-dependent protein kinase 5 (cdk5) [2], [3], [4], [5] as well as protein phosphatase 2A (PP2A) [6], [7], [8], [9], [10]. A down-regulation of PP2A in AD brain was found by our and other groups [9], [11], [12], [13], [14], suggesting that this decrease may be partially responsible for the abnormal hyperphosphorylation of tau in AD.

It was demonstrated recently that tau phosphorylation is negatively regulated by O-GlcNAcylation, a posttranslational modification of proteins with β-N-acetylglucosamine (GlcNAc) [15], [16], [17], [18], [19]. Like protein phosphorylation, O-GlcNAcylation is dynamically regulated by O-GlcNAc transferase (OGT), the enzyme catalyzing the transfer of GlcNAc from UDP-GlcNAc donor onto proteins, and N-acetylglucosaminidase (OGA), the enzyme catalyzing the removal of GlcNAc from proteins [20]. Global O-GlcNAcylation and specifically tau O-GlcNAcylation is decreased in AD brain [19]. These observations suggest that decreased brain glucose metabolism may promote abnormal hyperphosphorylation of tau via down-regulation of O-GlcNAcylation, a sensor of intracellular glucose metabolism [21]. However, tau is abnormally hyperphosphorylated at multiple phosphorylation sites and phosphorylation at various sites has different impacts on tau function and pathology [22]. How O-GlcNAcylation affects site-specific tau phosphorylation in vivo is not well understood [23].

In this study, we injected a highly selective OGA inhibitor, thiamet-G, into the lateral ventricle of mice to increase O-GlcNAcylation of proteins and investigated alterations of site-specific tau phosphorylation. We found that acute high-dose thiamet-G treatment led to decreased phosphorylation at some sites but increased phosphorylation at other sites of tau in the brain. We further investigated possible underlying mechanisms for these differential effects.

## Materials and Methods

### Antibodies and Reagents

The primary antibodies used in this study are listed in Table 1. Peroxidase-conjugated anti-mouse and anti-rabbit IgG were obtained from Jackson ImmunoResearch Laboratories (West Grove, PA, USA). The enhanced chemiluminescence (ECL) kit was from Amersham Pharmacia (Piscataway, NJ, USA). Thiamet-G was synthesized as described previously [23]. Other chemicals were from Sigma (St. Louis, MO, USA).

**Table 1:** Primary antibodies employed in this study.

Antibody	Type	Specificity	Phosphorylation sites	Reference/Source
RL2	Mono-	O-GlcNAc		Affinity Bioreagents, Golden, CO, USA
92e	Poly-	Tau		[44]
pT188	Poly-	P-tau	Thr181	Invitrogen, Carlsbad, CA, USA
pS199	Poly-	P-tau	Ser199	Invitrogen
pS202	Poly-	P-tau	Ser202	Invitrogen
pT205	Poly-	P-tau	Thr205	Invitrogen
pT212	Poly-	P-tau	Thr212	Invitrogen
pS214	Poly-	P-tau	Ser214	Invitrogen
pT217	Poly-	P-tau	Thr217	Invitrogen
pS262	Poly-	P-tau	Ser262	Invitrogen
pS356	Poly-	P-tau	Ser356	Invitrogen
pS396	Poly-	P-tau	Ser396	Invitrogen
pS404	Poly-	P-tau	Ser404	Invitrogen
pS409	Poly-	P-tau	Ser409	Invitrogen
pS422 (R145)	Poly-	P-tau	Ser422	[44]
Anti-p-GSK-3β	Poly-	P-GSK-3β	Ser9	Cell Signaling Technology, MA, USA
Anti-p-GSK-3β	Poly-	P-GSK-3β	Tyr216	Invitrogen
R133d	Poly-	GSK-3β		[45]
Anti-p-AKT	Poly-	P-AKT	Ser473	Cell Signaling Technology
Anti-AKT	Poly-	AKT		Cell Signaling Technology
Anti-p-PI3K (85 kDa)	Poly-	P-PI3K (85 kDa)	Tyr458/Tyr199	Cell Signaling Technology
Anti-PI3K (85 kDa)	Poly-	PI3K (85 kDa)		Cell Signaling Technology
Anti-CDK5	Poly-	CDK5		Santa Cruz Biotechnology, CA, USA
Anti-p35	Poly-	p35		Santa Cruz Biotechnology
Anti-GAPDH	Mono-	GAPDH		Santa Cruz Biotechnology

### Animals and Intracerebroventricular (icv) Injection

Thirty transgenic (Tg) mice (male, ∼6 months old) that express the largest isoform of wild-type human tau, tau_441_, were used in this study. The transgenic mice [24] were originally from Dr. A. Takashima of the Riken Brain Science Institute, Saitama, Japan, and were bred in our institute's animal colony. The mice were housed in a temperature-controlled room and fed standard rodent food pellets and water. The use of animals was in accordance with the guidelines of the National Institutes of Health and was approved by the Animal Welfare Committee of the New York State Institute for Basic Research in Developmental Disabilities.

**Figure 1:**
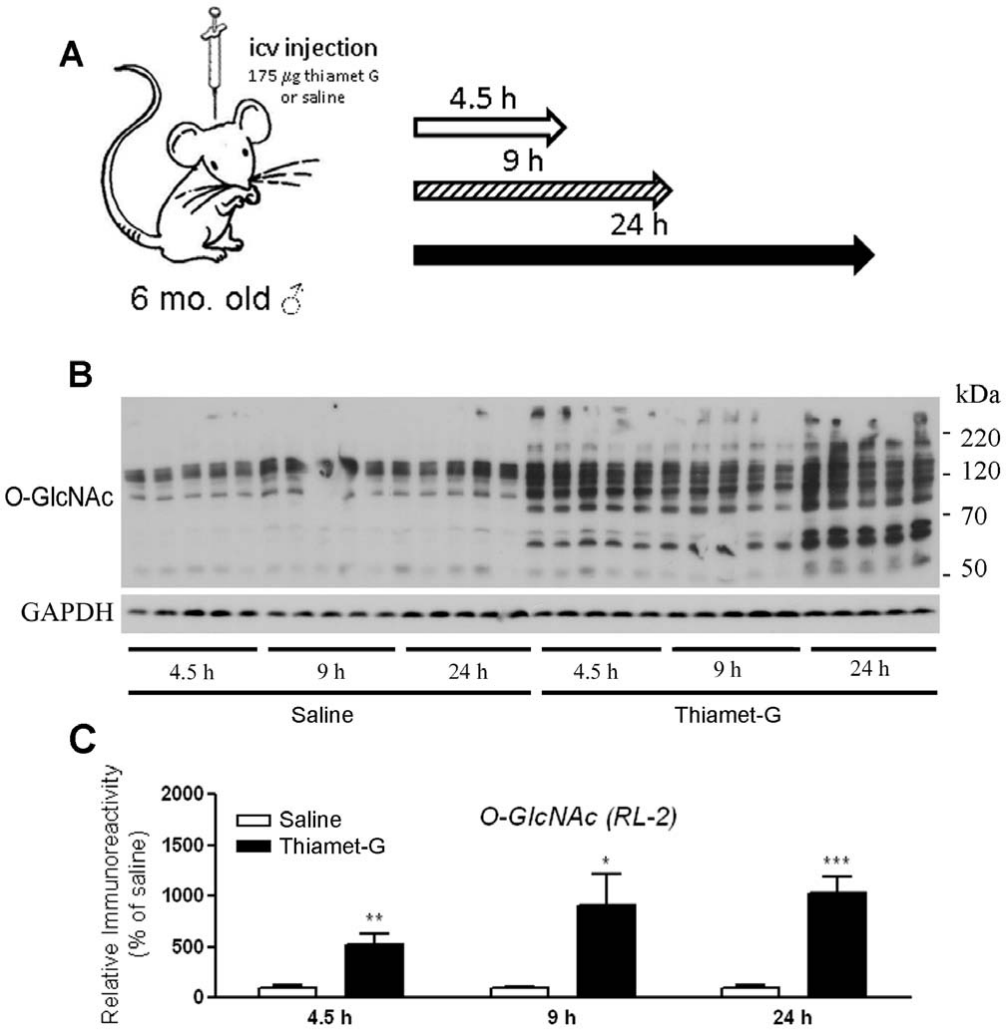
Experimental design and effects of icv injection of thiamet-G on brain protein O-GlcNAcylation. (A) Diagram showing the experimental design of icv injection. Thiamet-G (175 µg per mouse) was injected into the lateral ventricles of the brain, and then the tau Tg mice were sacrificed 4.5 h, 9 h, or 24 h after injection. (B) The brain homogenates were analyzed by Western blots developed with monoclonal antibody RL2 against O-GlcNAcylated proteins and, as a loading control, anti-GAPDH. (C) All of the RL2-positive bands in the blots were quantified densitometrically, and the intensities of the total O-GlcNAcylated proteins after being normalized by the intensity of GAPDH are shown (mean ± SEM; n = 5; *, *p*<0.05; **, *p*<0.01; ***, *p*<0.001 vs. saline controls).

Mice were first anesthetized by an intraperitoneal injection of 0.3% sodium pentobarbital (40 mg/kg). Thiamet-G (2.5 µl of 35 µg/µl) dissolved in 0.9% NaCl was injected bilaterally into the lateral ventricles of the brains at a dose of 175 µg/mouse, which correlated to a final concentration of approximately 700 µM in the brain, assuming a combined brain and spinal cord volume of 900 µL. The same volume of 0.9% NaCl alone was injected in control mice. The stereotaxic coordinates for the icv injection were: 0.4 mm anterior to posterior Bregma, 1.0 mm mid to lateral, and 2.2 mm dorsal to ventral dura. Mice were euthanized by cervical dislocation 4.5 h, 9 h or 24 h after icv injection (Fig. 1A). Anesthesia was not used to sacrifice mice because it affects tau phosphorylation [25], [26]. The brains were removed and stored at −80°C immediately for processing at a later time.

### Western Blot Analysis

The left hemispheres of the mouse brains were homogenized in pre-chilled buffer containing 50 mM tris-HCl (pH7.4), 8.5% sucrose, 10 mM β- mercaptoethanol, 1.0 mM EGTA, 1.0 mM phenyl-methylsulfonyl fluoride, 2.0 µg/ml aprotinin, 25 µg/ml leupeptin, and 1.0 µg/ml pepstatin. Protein concentrations of the homogenates were determined by modified Lowry method [27]. The samples were resolved in 10% SDS-PAGE and electrotransferred onto Immobilon-P membrane (Millipore, Bedford, MA, USA). The blots were then probed with primary antibodies and developed with the corresponding horseradish peroxidase–conjugated secondary antibody and enhanced chemiluminescence kit (Pierce, Rockford, IL, USA). Densitometric quantification of protein bands in Western blots was analyzed by using TINA software (Raytest IsotopenmeBgerate GmbH, Straubenhardt, Germany). Quantitative comparisons were analyzed by using student *t*-test, and the differences between groups were regarded to be significant when *p*<0.05.

### Culture of Adult Hippocampal Progenitor (AHP) Cells and PC12 cells

Neuronal progenitor cells were isolated from the hippocampus of adult Wistar rats (Charles River Laboratories, Wilmington, MA, USA) and cultured in neurobasal A medium consisting of 2% B27, 0.5 mM L-glutamine, 100 units/ml penicillin, 100 µg/ml streptomycin, and 10 ng/ml fibroblast growth factor-2 (FGF-2) at 37°C in a humidified 5% CO_2_ atmosphere, as described previously [28]. The medium was routinely changed every another day. When the cells reached ∼80% confluence, they were treated with 20 nM thiamet-G for 0.5–24 hr. At the end of the treatments, the cells were washed with PBS three times and then lysed by using Lammlie SDS-gel sample buffer that also contained 50 mM NaF, 1.0 mM Na_3_VO_4_, 1.0 mM phenyl-methylsulfonyl fluoride, 2.0 µg/ml aprotinin, 25 µg/ml leupeptin and 1.0 µg/ml pepstatin.

Differentiated AHP cells were also used in this study. The AHP cells were differentiated by replacing FGF-2 with 5 µM retinoic acid and 10% fetal calf serum when the cells reached approximately 80% confluency. The neuronal cultures were treated with 20 nM thiamet-G after 5–7 days of growth in the differentiation medium. At this stage, approximately 80% of the cells expressed the neuronal markers βIII-tubulin and microtubule-associated protein 2, and less than 5% expressed the astroglial marker Glial fibrillary acidic protein or the oligodendrocyte marker O4 (data not shown).

PC12 cells that stably express the largest isoform of human brain tau, tau_441_, were generated as described [16]. The cells were differentiated with 50 ng/ml nerve growth factor for 6 days before treatment with thiamet-G.

### Statistical analyses

Where appropriate, the data are presented as mean ± SEM. One-way ANOVA and post hoc analyses were performed using the scientific statistic software Graph Pad Prism 4 (San Diego, CA, USA). *p*<0.05 was considered to be statistically significant.

## Results

### Thiamet-G increases protein O-GlcNAcylation level in the brain

To investigate the effects of thiamet-G on tau phosphorylation at multiple phosphorylation sites in vivo, we first confirmed the elevation of protein O-GlcNAcylation level of the brains of tau Tg mice after icv injection of the drug. We observed a time-dependent elevation of O-GlcNAcylation after thiamet-G treatment, as determined by Western blots developed with monoclonal antibody RL2 toward O-GlcNAc (Fig. 1B). A marked increase in O-GlcNAcylation of numerous proteins at the range from 50-kDa to larger than 220 kDa was seen. Quantification of these RL2 positive bands indicated a 5-fold increase in global O-GlcNAcylation 4.5 h after thiamet-G injection and a 10-fold increase 24 h after injection (Fig. 1C). These results indicate a marked increase in O-GlcNAcylation in the mouse brains after Tiamet-G injection.

### Thiamet-G induces site-specific changes of tau phosphorylation in the mouse brain

**Figure 2:**
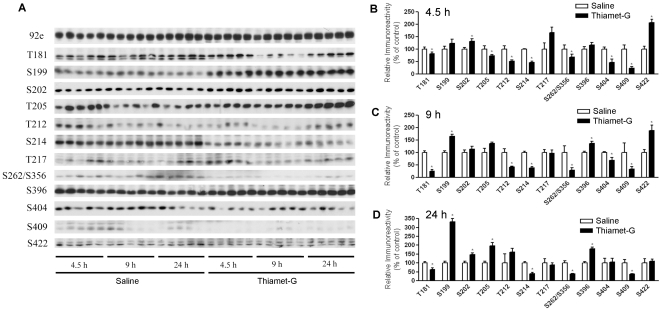
Levels of phosphorylation of tau at different sites in tau Tg mouse brains after icv injection of thiamet-G. (A) Western blots of the brain homogenates developed with an antibody to total tau (92e) and a battery of phosphorylation-dependent and site-specific antibodies to determine tau phosphorylation at individual phosphorylation sites, as indicated at the left side of the blots. (B–D) Levels of tau phosphorylation at individual sites 4.5 h (B), 9 h (C), or 24 h (D) after injection were determined by densitometrical quantitation of the blots, as shown in A. Data (mean ± SEM; n = 5) are presented as percentage of saline control injection. The total tau level of each sample was used for normalization for calculation. *p<0.05 vs. saline injection.

We then studied alterations of tau phosphorylation at individual phosphorylation sites in the mouse brains after treatment with thiamet-G by using Western blots developed with a battery of phosphorylation-dependent and site-specific tau antibodies. Antibody 92e toward total tau in a phosphorylation-independent manner was also included to check whether there was any change in the total tau level. We found that thiamet-G did not alter tau level in the mouse brain, but led to site-specific, bi-directional changes of tau phosphorylation (Fig. 2). Along with the marked increase in O-GlcNAcylation after thiamet-G treatment, we observed decreased tau phosphorylation at Thr181, Thr212, Ser214, Ser262/Ser356, Ser404 and Ser409. In contrast, tau phosphorylation was increased instead at some other sites such as Ser199, Ser202, Ser396 and Ser422 upon thiamet-G treatment. Among the 12 phosphorylation sites studied, tau phosphorylation at only one site (Thr217) was not significantly changed upon thiamet-G treatment. Whereas the alterations of tau phosphorylation at Thr205 was found to be bi-directional, with a decrease 4.5 h after thiamet-G injection and an increase 9 and 24 h after thiamet-G injection. These results indicate that the effects of thiamet-G–induced tau phosphorylation are not only dynamic, but also site-specific and bidirectional.

### Thiamet-G induces activation of GSK-3β in the mouse brain

**Figure 3:**
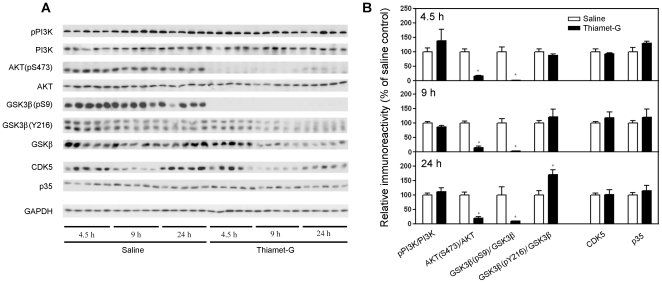
Analyses of major tau kinases in tau Tg mouse brains after thiamet-G treatments. (A) Western blots of the brain homogenates developed with antibodies indicated at the left side of the blots. GAPDH blot was included as a loading control. (B) The blots were quantified densitometrically, and the immunoreactivities (mean ± SEM; n = 5) of the indicated phosphorylated kinases over that of the total kinase counterparts are shown to represent the activation statuses of these kinases. Note that the GSK-3β(Y216) antibody also recognized GSK-3α (upper band in A). Only the GSK-3β band (lower band) was used for quantification. In case of CDK and p35, GAPDH was used for normalization. *p<0.05 vs. saline injection.

Negative regulation of phosphorylation by O-GlcNAcylation of tau has been observed previously [15], [16], [18], [19], [23]. To investigate why tau phosphorylation at Ser199, Ser202, Ser396 and Ser422 was increased when O-GlcNAcylation was elevated upon thiamet-G treatment, we studied the major tau kinases. We first studied GSK-3β and its upstream regulating pathway, the PI3K-AKT signaling pathway. GSK-3β activity is mainly regulated negatively via its phosphorylation at Ser9 by AKT, which in turn is regulated by PI3K and other factors [29]. We found that thiamet-G treatment did not significantly alter the level of GSK-3β except after treatment for 9–24 h, but blocked its phosphorylation at Ser9 completely (Fig. 3A), suggesting that GSK-3β was markedly activated under these conditions. Phosphorylation of GSK-3β at Tyr216, which makes it more active, was also increased 24 h after thiamet-G treatment. Consistent with the almost complete dephosphorylation of GSK-3β at Ser9, Ser473 and Thr308 phosphorylation of AKT, which determines its kinase activity, was also blocked by thiamet-G treatment. However, we did not find any significant changes of either the level or the activation of PI3K, the major upstream kinase of AKT, in the mouse brain after thiamet-G treatment. These results suggest that thiamet-G induced over-activation of GSK-3β via inhibition of AKT phosphorylation. The over-activation of GSK-3β may explain the increased tau phosphorylation at Ser199, Ser202, Ser396 and Ser422, because these sites are the phosphorylation sites catalyzed mainly by GSK-3β [30].

CDK5 is the second most important tau kinase in the brain [31] and is activated by p35/p25. We thus also studied the level of CDK5 and its activators. We found that thiamet-G did not alter either CDK5 or p35 (Fig. 3). P25, which is the truncated and a more active form of p35, was not detectable in the mouse brain.

### Thiamet-G induces increased O-GlcNAcylation, but fails to activate GSK3β or increase tau phosphorylation in cultured neuronal cells

**Figure 4:**
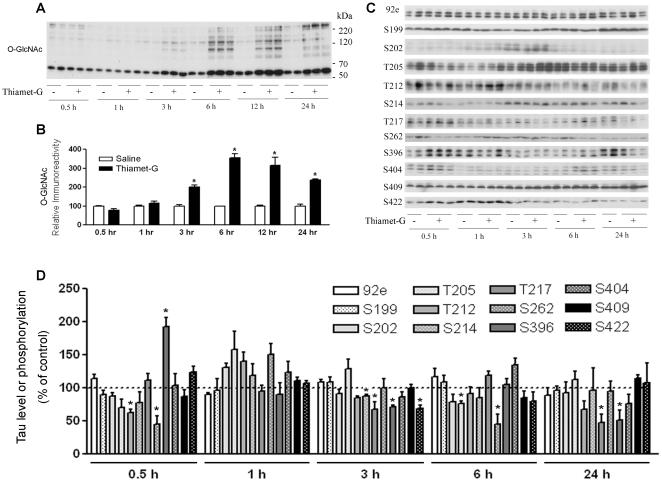
Effects of thiamet-G treatment on protein O-GlcNAcylation and tau phosphorylation in AHP cells. (A) Proliferating AHP cells were treated with 20 nM thiamet-G for various periods of time, and the protein O-GlcNAcylation of the cell lysates was determined by Western blots developed with RL-2 against O-GlcNAc. (B) The blots were quantified densitometrically, and the O-GlcNAc levels are presented as the percentage of control cells at each time point. (C) Western blots developed with 92e for detecting total tau level and with several phosphorylation-dependent and site-specific tau antibodies for detecting phosphorylation of tau at individual sites. (D) The tau blots were quantified densitometrically, and the tau level and site specific phosphorylation level are presented as the percentage of control cells at each time point. The data shown are mean ± SEM of triplicates of one of three separate experiments with similar results.

To elucidate the complex regulation of site-specific phosphorylation induced by thiamet-G, we employed cultured cells, because cell cultures are more easily manipulated. We first selected AHP cells because they are more close to brain neurons than tumor cell lines. As expected, treatment of AHP cells with 20 nM thiamet-G increased protein O-GlcNAcylation (Fig. 4A). While we observed decreased tau phosphorylation at several phosphorylation sites in the AHP cells after thiamet-G treatment, it did not induce any significant increase in tau phosphorylation at the phosphorylation sites studied except at a transient elevation of Ser396 at 30 min after the treatment (Fig. 4C, D). We then investigated the levels and the activation of GSK-3β and the upstream PI3K-AKT signaling transduction pathway, as well as CDK5/p35. We did not find any significant changes upon the treatments with thiamet-G in AHP cells (data not shown). These results further support our conclusion above that the increased tau phosphorylation at some sites observed in the thiamet-G treated mouse brains was due to GSK3β activation.

To learn whether the phenomena we observed in undifferentiated AHP cells were specific to these cells, we also performed similar experiments in differentiated AHP cells and differentiated PC12 cells. As seen in proliferating AHP cells, we did not observe any marked elevation of tau phosphorylation at any phosphorylation sites or changes of tau kinases upon thiamet-G treatments in these two types of cells (data not shown).

## Discussion

Thiamet-G is a highly specific OGA inhibitor that was synthesized based on rationale design [23]. Initial studies indicated that this compound reduce tau phosphorylation at some phosphorylation sites that can be abnormally phosphorylated in AD [23], suggesting that OGA inhibition may offer a potential therapeutic approach for slowing tau-mediated neurodegeneration seen in AD and other tauopathies. Because tau phosphorylation at different sites has different impacts on tau's function and pathology [22], investigating the role of thiamet-G on site-specific tau phosphorylation is needed. In this study we found that icv injection of thiamet-G led to decreased tau phosphorylation at Thr181, Thr212, Ser214, Ser262/Ser356, Ser404 and Ser409, but increased tau phosphorylation at Ser199, Ser202, Ser396 and Ser422 in the mouse brain.

We and other groups have previously shown that O-GlcNAcylation regulates phosphorylation of tau in a reciprocal manner overall [15], [16], [17], [18], [19], [23]. Our observations of decreased tau phosphorylation at Thr181, Thr212, Ser214, Ser262/Ser356, Ser404 and Ser409 are consistent with previous studies, further supporting the reciprocal relationship between O-GlcNAcylation and phosphorylation of tau at these sites. To understand the underlying mechanism by which thiamet-G increased tau phosphorylation at some other phosphorylation sites in the mouse brain, we investigated the effects of thiamet-G injection on the major tau kinases. We found that thiamet-G at the dose injected into the brain completely blocked Ser9 phosphorylation of GSK-3β, indicating a marked activation of this major tau kinase. Our previous studies have demonstrated that tau phosphorylation at Ser199, Ser202 and Ser396 are mainly regulated by GSK-3β [30]. Taken together, we conclude that the elevation of tau phosphorylation at Ser199, Ser202, Ser396 and Ser422 results from the combinational effects of the increase in tau O-GlcNAcylation and the activation of GSK-3β activity, and that GSK-3β activation predominated and led to the net increase in tau phosphorylation at these sites in the mouse brain. Our results showed that GSK-3β was not activated in cultured neuronal cells treated with thiamet-G, consistent with the absence of any increase in tau phosphorylation at these phosphorylation sites. In a previous study, when thiamet-G was administered to rats orally for 24 hrs, tau phosphorylation at these sites was not found to be increased [23]. Whether the discrepancy between this previous study and the present study is due to different routes of drug administration (oral vs. icv injection), the attainment of different doses within the brain, or the use of different species (rat vs. mouse) is currently unknown. It is possible that there is either a dose-dependent effect of thiamet-G on GSK-3β–stimulation or an off target effect of thiamet-G when used at high doses directly in the brain. Indeed, it is likely that the icv injection of this study led to a much higher thiamet-G concentration in the central nervous system than that from oral dosing. Unfortunately, GSK-3β modification and activity in the brain was not examined in the previous study, so direct comparisons are not possible.

Our results indicated marked differences in the effects of thiamet-G on tau phosphorylation between the mouse brains and the cultured neurons. Further experiments indicated that thiamet-G–induced increase of tau phosphorylation at several sites resulted from activation of GSK-3β, a major tau kinase, but this activation did not occur in cultured cells. Different regulations of tau phosphorylation by extracellular signaling between the brains and the cultured neurons might also contribute to the different results we observed. A previous study has demonstrated that tau phosphorylation is regulated by FGF-2 through GSK-3β [32]. Our observations of GSK-3β activation in the brains but not in the cultured cells are consistent with this speculation. The mice we used for this study expressed the wild-type human tau isoform. Although we cannot absolutely rule out the possibility that the different results between the mouse brains and the cell cultures might also result from different tau species (mouse/human tau versus rat tau), this possibility is very small, because our observations that thiamet-G–induced GSK-3β activation in the brains, but not in the cultured cells, can explain the different results in these two systems nicely.

Our studies on the upstream regulating kinases of GSK-3β suggest that thiamet-G led to marked GSK-3β activation as the result of an inhibition of AKT by reducing its phosphorylation at Ser473 and Thr 308, which regulates its activity positively [29]. AKT inhibition may also contribute to the thiamet-G–induced decrease of tau phosphorylation at Thr212 and Ser214, because these two sites are also substrates of AKT [33]. AKT phosphorylation was mainly catalyzed by the mTORC2 complex and PI3K–phosphoinositide-dependent protein kinase-1 (PDK1). Because we did not observe the corresponding decrease in PI3K, we speculate that the reduction/elimination of phosphorylation of AKT and GSK-3β after thiamet-G treatment may result from elevation of O-GlcNAcylation of AKT, PDK1 and/or mTOR. Alternatively, it could be off-target effect of the inhibitor when used at high doses. Phosphorylation of these kinases has been reported to be regulated negatively by O-GlcNAcylation [34], [35], [36], [37]. It is worth noting that different effects of OGA inhibition on phosphorylation of AKT and GSK-3 have been reported. Elevation of O-GlcNAcylation in skeletal muscles after OGA inhibition using another inhibitor, PUGNAc, does not significantly alter insulin-stimulated phosphorylation of AKT or GSK-3 [38]. In differentiated 3T3-L1 adipocytes, two different OGA inhibitors have been found to increase O-GlcNAc levels but not alter insulin-stimulated phosphorylation of AKT nor induce insulin resistance either [39], [40]. Therefore, it remains somewhat unclear as to the effects of OGA inhibition on alteration of GSK-3β levels and AKT activity; the effects observed here could stem from high-dose inhibition of OGA, or alternatively from off-target effects of using the inhibitor at a high dose.

Tau is abnormally hyperphosphorylated and aggregated in AD and other tauopathies. Previous studies from our and other groups have demonstrated differential roles of tau phosphorylation at various phosphorylation sites. A quantitative in vitro study demonstrated that phosphorylation of tau at Ser262, Thr231, and Ser235 inhibits its binding to microtubules by ∼35%, ∼25%, and ∼10%, respectively [41]. In vitro kinetic studies of the binding between hyperphosphorylated tau and normal tau suggest that Ser199/Ser202/Thr205, Thr212, Thr231/Ser235, Ser262/Ser356 and Ser422 are among the critical phosphorylation sites that convert tau to an inhibitory molecule that sequesters normal microtubule-associated proteins from microtubules [42]. Further phosphorylation at Thr231, Ser396, and Ser422 promotes self-aggregation of tau into filaments. It is obvious that tau phosphorylation at various sites impacts tau activity and aggregation collectively. Our recent study has demonstrated that tau phosphorylation at the proline-rich region, which is located upstream of the microtubule-binding domains, inhibits its microtubule assembly activity moderately and promotes its self-aggregation slightly. Tau phosphorylation at the C-terminal tail region increases its activity and promotes its self-aggregation markedly. Tau phosphorylation at both of these regions plus the microtubule-binding region nearly diminishes its activity and disrupts microtubules [43]. Therefore, the overall impacts of thiamet-G on tau need to be further verified by its functional studies, and testing the effects of thiamet-G on cognitive function in mouse AD models, especially using different doses, becomes urgent before considering it to be a therapeutic agent for treating AD.

In conclusion, thiamet-G is a specific OGA inhibitor and is very effective in elevating protein O-GlcNAcylation level in the mammalian brain. Because thiamet-G not only directly modulated tau phosphorylation inversely, but also stimulated GSK-3β activity likely via inhibition of AKT, the final consequences were a decrease in tau phosphorylation at Thr181, Thr212, Ser214, Ser262/Ser356, Ser404 and Ser409, and an increased in tau phosphorylation at Ser199, Ser202, Ser396 and Ser422 in the brain after thiamet-G treatment. The site-specific, bi-directional regulation of tau phosphorylation warrants further studies on evaluation of dose and time dependent effects on OGA inhibition.
